# Characteristics and Stability of Valerian Essential Oil Nanoemulsion: A Comparison Between Ultrasonication and Microfluidization

**DOI:** 10.1002/fsn3.71959

**Published:** 2026-06-02

**Authors:** Azizeh Rezaei, Zohreh Hamidi‐Esfahani, Ali Sedaghat Doost, Paul Van der Meeren

**Affiliations:** ^1^ Department of Food Science and Technology, Faculty of Agriculture Tarbiat Modares University Tehran Iran; ^2^ Particle and Interfacial Technology Group (PaInT), Department of Green Chemistry and Technology, Faculty of Bioscience Engineering Ghent University Gent Belgium

**Keywords:** entrapment efficiency, Lumisizer, microfluidization, nanoemulsion, ultrasonication, valerian essential oil

## Abstract

Nanoemulsions are increasingly recognized as promising delivery systems for essential oils in functional products due to their ability to protect sensitive bioactives. Valerian essential oil (VEO) contains valuable bioactives with notable functional properties; however, its volatility and instability limit its direct incorporation into aqueous environments. This study investigated the preparation of oil‐in‐water nanoemulsions containing 1.5% (w/w) VEO using two high‐energy emulsification techniques, ultrasonication (US) and microfluidization (MF), in phosphate buffer (PB) or distilled water (DW) as dispersion media. Samples were characterized in terms of particle size, polydispersity index (PDI), zeta potential, entrapment efficiency, and physical stability. The most stable sample was further evaluated under environmental stress, including varying ionic strength (up to 150 mM NaCl) and pH (4–8). In the formulation prepared in PB using US (US‐PB), smaller particle sizes were observed compared to those prepared using MF (MF‐PB) (136.4 ± 0.12 vs. 146.5 ± 0.26 nm), showing a similar trend in both formulations prepared in DW (US‐DW and MF‐DW) (139 ± 4.57 vs. 142.4 ± 0.47 nm). All samples remained monodispersed (PDI < 0.3). Zeta potential values indicated stronger stabilization in formulations prepared in PB (−56.89 ± 1.61 mV for US‐PB and −50.16 ± 5.34 mV for MF‐PB) compared with DW (−22.93 ± 5.68 mV for US‐DW and −26.17 ± 7.90 mV for MF‐DW). Lumisizer analysis showed slightly higher stability for US‐PB (instability index 0.37) compared with MF‐PB (0.40), whereas DW systems were less stable (0.67 and 0.54 for US‐DW and MF‐DW, respectively). Formulations prepared in PB, particularly US‐PB exhibited the highest entrapment efficiency (> 88%). At 150 mM NaCl concentration and pH 4.0, US‐PB stability decreased due to reduced zeta potential and electrostatic repulsion, whereas it remained stable at pH 8.0.

## Graphical abstract

The graphical abstract illustrates the preparation and stability evaluation of valerian essential oil (VEO) nanoemulsion produced using ultrasonication and microfluidization in phosphate buffer (PB) and distilled water (DW). Both high‐energy methods successfully generated nano‐sized emulsions; however, ultrasonication produced smaller droplet sizes and narrower particle size distributions than microfluidization. Nanoemulsions prepared in PB exhibited superior physicochemical stability, resistant to creaming and phase separation during 35 days of storage. In contrast, formulations prepared in DW showed lower electrostatic stability, pH reduction, droplet aggregation, and visible oiling‐off over time. Environmental stress testing showed that increasing NaCl concentration and acidic pH reduced surface charge and destabilized the emulsions, whereas alkaline pH preserved stability. Among all formulations, the ultrasonicated nanoemulsion in phosphate buffer showed the highest entrapment efficiency and best overall long‐term stability under different storage and environmental conditions.

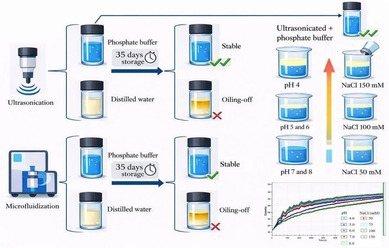

## 1 Introduction

The growing consumer demand for “clean label” (Sedaghat Doost, Nikbakht Nasrabadi, Kassozi, et al. 2020), functional (Rezaei et al. 2019) products and natural preservatives (Sahraei et al. 2025) has reshaped the food and pharmaceutical industries. With increasing awareness of the risks associated with synthetic and conventional chemical additives, researchers and manufacturers are turning toward natural alternatives with preservative, functional, and therapeutic benefits (Rezaei et al. 2019; Sedaghat Doost et al. 2018). Among these, plant‐derived essential oils (EOs) have attracted considerable attention due to their rich bioactive compounds, broad biological activity, and potential to enhance food quality and human health (Tavakoli et al. 2023).

Valerian ( *Valeriana officinalis* L.) is one of the most historically significant medicinal plants used for sleep and anxiety disorders. It is valued for its sedative, anxiolytic, analgesic, anticonvulsant, and neuroprotective properties in both humans and animals (Ebrahimie et al. 2015; Nandhini et al. 2018; Wang et al. 2020). It is classified by the FDA (21 CER 172.510) as a natural substance used as a flavoring agent. Recent studies highlight its antimicrobial activity and potential role as a natural bio‐preservation in clean‐lable products (Das et al. 2023). Valerian root contains 0.3%–0.8% EO and more than 150–200 bioactive constituents, including valeric acid, valepotriates, flavonoids, triterpenes, lignans, alkaloids, and a broad spectrum of sesquiterpenes (Chen et al. 2015; Sermukhamedova et al. 2017). These compounds collectively contribute to its beneficial effects, making valerian essential oil (VEO) a promising candidate for clean‐label food developments.

However, directly incorporating EOs into food systems presents challenges. Many EOs possess intense aromas, bitter tastes, dark colors, and chemical instability. Their lipophilic nature and susceptibility to oxidation, polymerization, and interactions with other food components further complicate their incorporation (Dillard and Bruce German 2000; Marzieh et al. 2013). To overcome these limitations, incorporation technologies, particularly liposomal and nanoemulsion systems (Zhou et al. 2018), play a critical role in improving EO stability, masking undesirable sensory attributes, and enhancing bioavailability (Ozdal et al. 2026; Sawale et al. 2017; Sedaghat Doost, Nikbakht Nasrabadi, Sadžak, et al. 2020).

Nanoemulsions are especially promising as carriers for hydrophobic EOs due to their biocompatibility, low toxicity, and structural resemblance to biological membranes. Typically stabilized by surfactants, including lecithin, nanoemulsion droplets consist of an internal oily phase surrounded by an interfacial layer that can entrap bioactive compounds and protect them from environmental degradation (Ozdal et al. 2026; Roy et al. 2016).

Several emulsification techniques exist for producing nanoemulsions, including ultrasonication and microfluidization. Ultrasonication is widely used due to its simplicity and ability to reduce particle size through high‐energy acoustic cavitation (Naeem et al. 2015; Ozdal et al. 2026; Putri et al. 2017). Microfluidization, on the other hand, employs intense shear and impact forces within a high‐pressure interaction chamber, yielding uniform and scalable dispersions. Both methods are solvent free; an advantage for food‐grade applications (Ozdal et al. 2026).

Choosing the optimal preparation method requires consideration of several factors: the physicochemical properties of the incorporated compound, the dispersion medium, the desired particle size and homogeneity, the stability requirements, and the intended application (Mozafari et al. 2008; Taylor et al. 2005). Moreover, parameters such as pH, ionic strength, lipid composition, and shear forces can influence particle size, zeta potential, and long‐term stability (Roy et al. 2016). In conclusion, method selection, sample preparation conditions, and physicochemical parameters such as pH and temperature have a strong influence on the long‐term stability of nanostructured delivery systems.

The present study aims to compare the physical properties and stability of VEO‐loaded nanoemulsions prepared using two common homogenization techniques, ultrasonication and microfluidization. Nanoemulsions containing 1.5% VEO were formulated in two different dispersion media (PB and DW) and characterized in terms of particle size, polydispersity index (PDI), zeta potential, entrapment efficiency (EE), and stability under centrifugal acceleration. Understanding how processing parameters and dispersion media affect nanoemulsion behavior will help optimize incorporation strategies for the development of clean‐label and functional food systems.

## 2 Materials and Methods

### 2.1 Materials

De‐oiled lecithin was purchased from Cargill (Belgium). VEO was bought from Pranarom (Belgium). Analytical grade chemicals were purchased from Sigma‐Aldrich Co (St. Louis, MO, USA).

### 2.2 Methods

#### 2.2.1 Preparation of o/w Nanoemulsions

##### 2.2.1.1 Preparation of Initial Emulsion

A 2% (w/w) lecithin stock solution was prepared separately in PB and DW. Sodium azide (0.02%) was incorporated to inhibit microbial contamination during storage and analysis. The mixture was stirred until fully hydrated and then stored at 4°C for subsequent use.

To prepare nanoemulsions containing VEO, 1.5% (w/w) EO was added to 0.5% lecithin solution. The mixture was pre‐emulsified using a high‐speed Ultra‐Turrax blender (Ultra‐Turrax, S 50 N–G 45 F, IKA‐Werke, Germany) at 24,000 rpm for 2 min. This step ensured the uniform distribution of the EO droplets and provided a suitable precursor for the formation of nano‐sized droplets (Sedaghat Doost, Van Camp, et al. 2019).

##### 2.2.1.2 Ultrasonication Process

Following Sedaghat Doost, Van Camp, et al. (2019) with slight changes, 30 mL of the coarse emulsion was transferred into a 40 mL PYREX low‐form beaker. The titanium horn probe (13 mm diameter) of the sonicator (Branson Sonifier S‐250A, USA) was immersed approximately 1 cm into the sample. Ultrasonication was performed at 70% amplitude for 4 min. Because ultrasonication generates substantial heat, potentially degrading EO components, the process was carried out under cooling conditions (10°C) using a cryo‐compact circulator (CF31, JULABO, Seelbach, Germany) to minimize thermal effects.

##### 2.2.1.3 Microfluidization Process

According to Huyst et al. (2025), with minor modifications, the 30 mL pre‐emulsion was processed through the Microfluidizer (M110‐S, Microfluidics Crop, Newton, MA) five times at 112 MPa, based on the preliminary investigation. Continuous cooling was applied to counteract heat accumulation due to high shear forces within the interaction chamber by placing the heat exchanger coil into an ice‐water batch. After processing, all samples were stored in glass tubes at 4°C for further characterization.

#### 2.2.2 Particle Size and PDI

*Z* average, PDI, and size distribution profiles were measured using a Zetasizer 3000HS (Malvern Instruments, UK) at 25°C. To prevent multiple scattering effects, samples were diluted (1:500) using the corresponding dispersion medium (PB or DW) before measurement. The refractive index of the dispersant was set to 1.331.

Measurements were conducted at a scattering angle of 90°, and the wavelength was set to 633 nm. Each reported value represents the mean ± standard deviation, obtained from three independent measurements, each averaged over 10 measurement runs. Monomodal analysis of the autocorrelation function was used to determine the size distribution. All results were reported as the means of three replications over a 35‐day storage period.

#### 2.2.3 Zeta Potential

Zeta potential (*ζ*) was determined using the electrophoretic mobility of droplets according to the Helmholtz‐Smoluchowski approximation. The viscosity and dielectric constant were set to values corresponding to water at 25°C (0.89 cP and 79.0), respectively. As with particle size analysis, samples were diluted with 10 mM KCl. The average of three independent measurements (each based on 10 runs) was reported as mean ± standard deviation.

#### 2.2.4 Stability and Creaming Behavior; pH and Ionic Strength Effect

Stability was assessed using a Lumisizer analytical centrifuge (Lumisizer 6512‐145, Berlin, Germany). Each sample was subjected to centrifugation at 4000 rpm and 25°C for different timings depending on the purpose, generating an accelerated stability profile equivalent to approximately some months under normal gravity. Transmission profiles were recovered every 60 s across the length of the cuvette (110–130 mm).

The Lumisizer software (SEPview 5.1) was used to calculate instability indices, which range from 0 (highly stable; no detectable separation) to 1 (fully unstable; complete phase separation). Lower instability index values correspond to more stable nanoemulsions. Changes in light transmission over time were interpreted as indicators of creaming or sedimentation.

The ionic strength was altered by adding NaCl at concentrations of 50, 75, 100, and 150 mM to the freshly prepared stable nanoemulsions. The pH was also adjusted to either acidic or alkaline levels (pH 4–8) using HCI and NaCl (both 0.1 mM). Samples were then kept at 4°C for 24 h before analysis.

#### 2.2.5 EE

The EE of the EO nanocarriers was determined using a UV–Vis spectrophotometer (VWR, Belgium) adapted from Sedaghat Doost, Kassozi, et al. (2019), with slight modifications. A measured volume of nanoparticle suspension was placed into Amicon centrifugal ultrafiltration tubes (Amicon Ultra, 10 kDa MWCO, 0.5 mL, Milipore, Cork, Ireland) and centrifuged at 10,000*g* for 30 min to separate the entrapped oil in droplets (retentate) from the free EO (permeate). The permeate was collected, diluted 22‐fold with methanol, and its absorbance was measured at 264 nm, corresponding to the characteristic secondary peak of the EO. A blank sample without EO, processed under identical conditions, was used for baseline correction. The concentration of free EO was determined using a calibration curve (Abs. = 0.0051C + 0.0358, *R* ^2^ = 0.9993) constructed from standard EO solutions (0–104 μg/mL) prepared from a 1.5% (w/w) EO stock in methanol. The EE% was calculated using the Equation (1):

(1) $$\begin{array}{c} \mathrm{EE}\;\left(\%\right)=\\ \left[\left(\text{Total}\;\mathrm{EO}\;\text{added}–\text{Free}\;\mathrm{EO}\;\text{in permeate}\right)/\text{Total}\;\mathrm{EO}\;\text{added}\right]\times 100 \end{array}$$

#### 2.2.6 Statistical Analysis

Data were analyzed using two‐way ANOVA, followed by Duncan's test at *p* < 0.05, using SPSS version 27.0. Results are shown as mean ± standard deviation from three replicates.

## 3 Results and Discussion

### 3.1 *Z* Average, Particle Size Distribution, and PDI

Particle size is one of the most important physicochemical properties for characterizing nanoemulsions. Smaller droplets typically offer improved stability, greater surface‐to‐volume ratio, and enhanced bioavailability of encapsulated bioactive compounds (Ozdal et al. 2026; Roy et al. 2016; Sedaghat Doost, Kassozi, et al. 2019). For these reasons, understanding how different production methods influence droplet size is crucial.

In this study, as shown in Table 1, microfluidization and ultrasonication, two high‐energy emulsification techniques, resulted in significant differences in particle sizes between the two dispersion media (*p* < 0.05). The strength of the mechanical force and the type of dispersion media are among the two factors that influence the stability of nanoemulsion (Ozdal et al. 2026). MF‐PB droplets exhibited a mean diameter of 146.5 ± 0.26 nm, whereas US‐PB droplets were smaller, at 136.4 ± 0.12 nm. A similar trend was observed in distilled water (DW), where 142.4 ± 0.47 and 139 ± 4.57 nm were measured for MF‐DW and US‐DW, respectively (*p* < 0.05) at Day 1. This indicates that ultrasonication, under the conditions used, produced more efficient size reduction than microfluidization. These findings align with the mechanism of acoustic cavitation during ultrasonication. The intense collapse of microbubbles produces strong localized shear and turbulence, which may more effectively break down lipid layers (Rajasekaran et al. 2024). However, high‐energy ultrasonication can also generate heat, stressing lipid components, which is why cooling was necessary throughout the process. All samples retained their nano‐scale particle size during storage time.

**TABLE 1:** *Z* average ± SD of nanoemulsions during storage time.

Sample	*Z* average (nm)					
	Day 1	Day 7	Day 14	Day 21	Day 28	Day 35
MF‐PB	146.5 ± 0.26^aA^	223.6 ± 0.95^aB^	256.9 ± 1.55^aC^	294.5 ± 0.86^aD^	350.2 ± 10.21^aE^	346.4 ± 0.45^aF^
US‐PB	136.4 ± 0.12^cA^	219.6 ± 1.21^bB^	252.7 ± 0.70^bC^	296.7 ± 0.93^aD^	355.5 ± 4.56^aE^	379.6 ± 3.16^bF^
MF‐DW	142.4 ± 0.47^bA^	153.2 ± 1.34^cB^	218.2 ± 14.23^cC^	141.6 ± 0.56^cA^	n.m.	n.m.
US‐DW	139.0 ± 4.57^bcA^	149.6 ± 0.35^dB^	180.0 ± 0.38^dC^	143.9 ± 1.96^cD^	n.m.	n.m.

*Note:* Different lowercase letters within the columns and different capital letters within the rows are significantly different (*p* < 0.05).

Abbreviations: DW, distilled water; MF, microfluidization; n.m., not measured due to instability; PB, phosphate buffer; US, ultrasonication.

**FIGURE 1:**
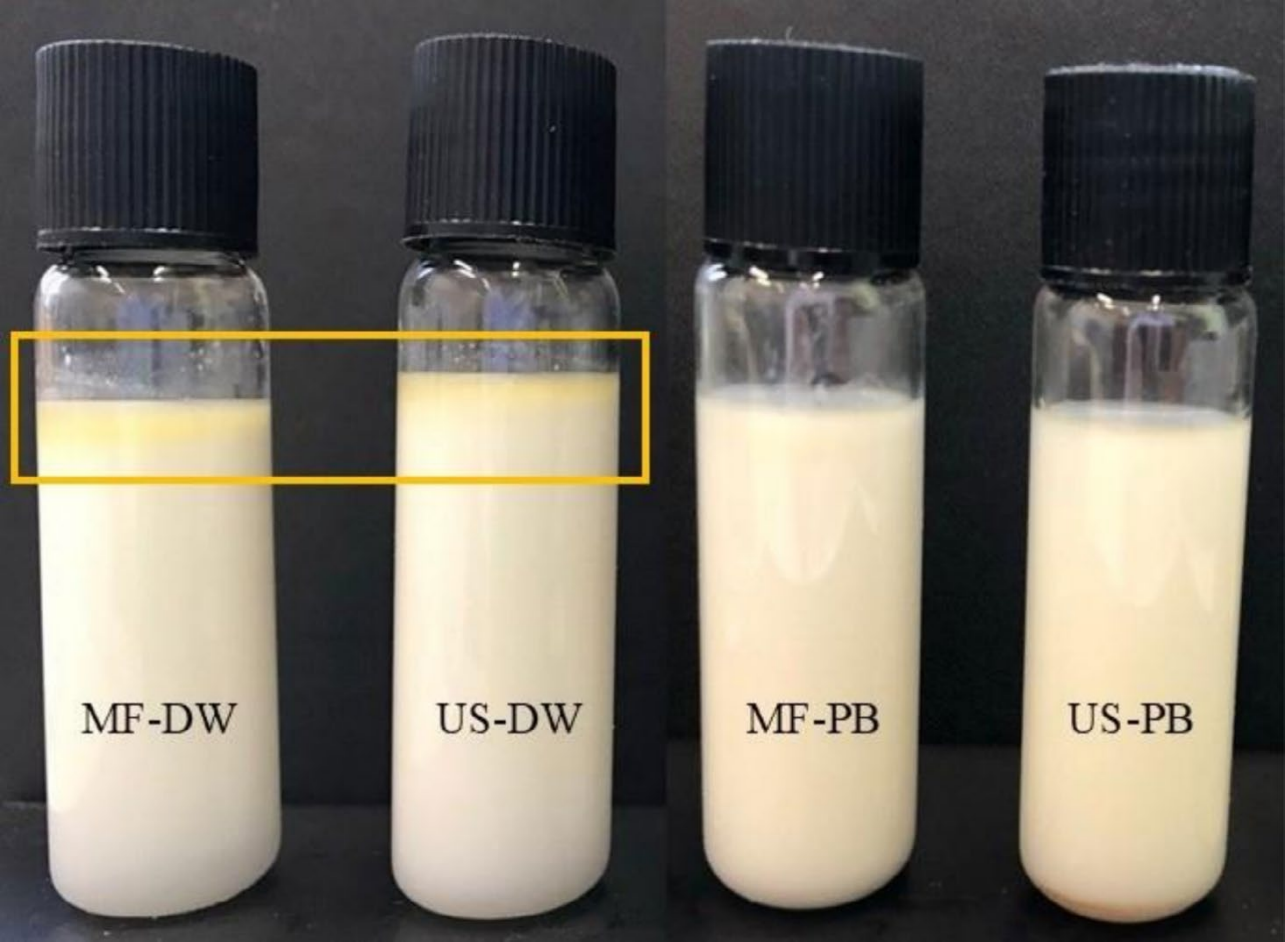
Visual inspection of samples during 35 days of storage.

**FIGURE 2:**
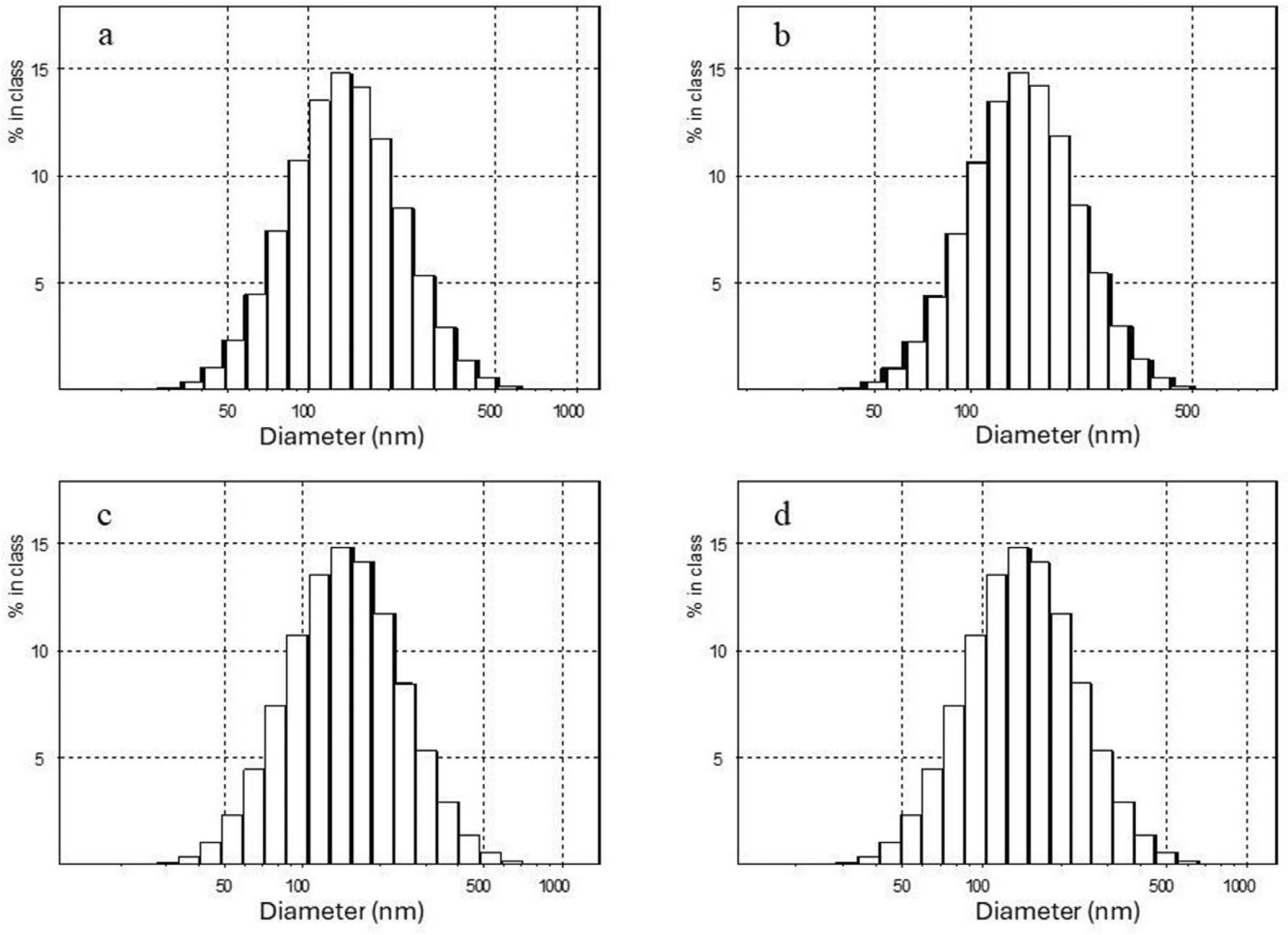
Particle size distribution of freshly prepared nanoemulsions (time zero): (a) US‐PB, (b) MF‐PB, (c) US‐DW, and (d) MF‐DW. DW, distilled water; MF, microfluidization; PB, phosphate buffer; US, ultrasonication.

**FIGURE 3:**
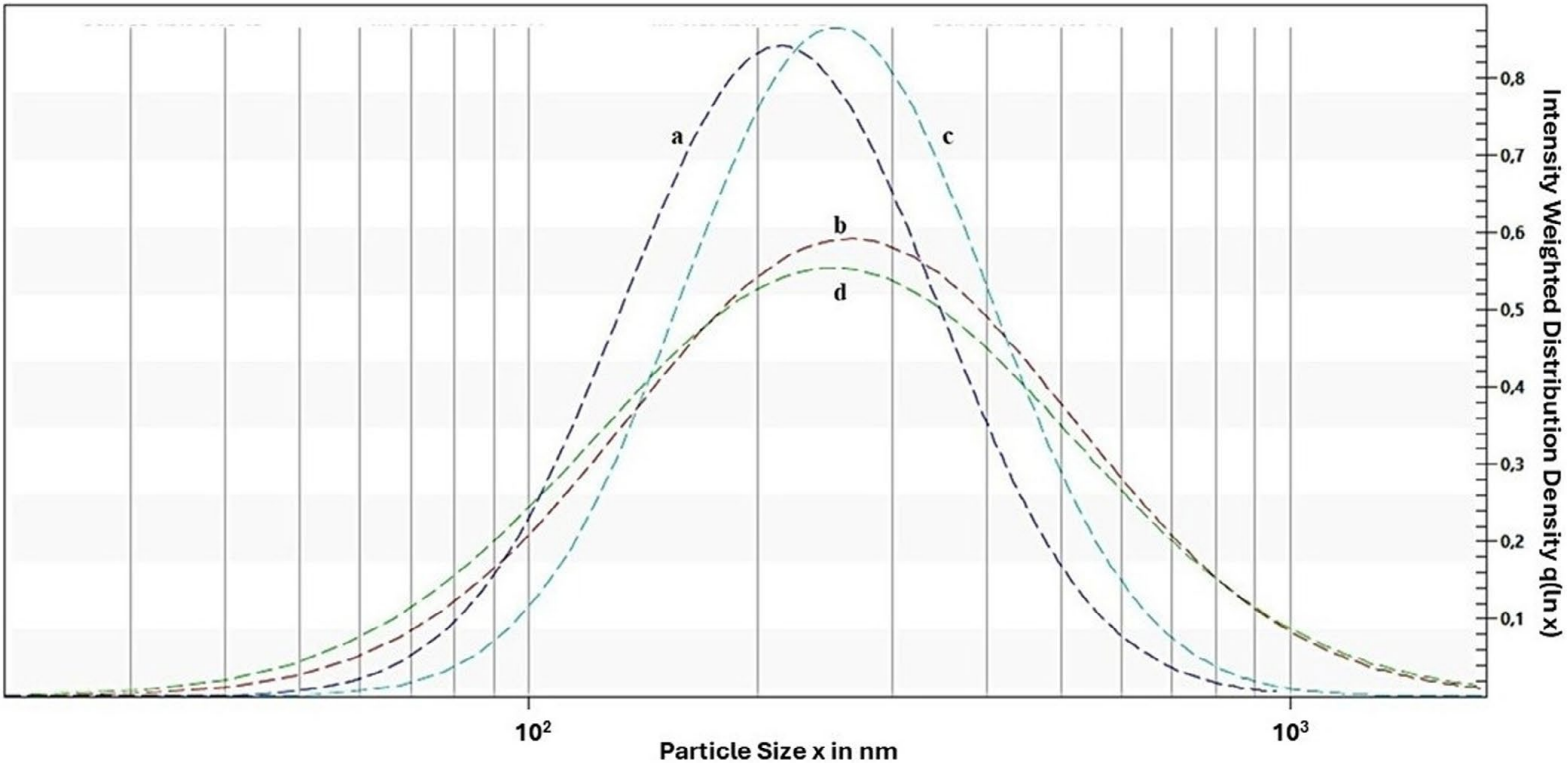
(Fitted) particle size distribution under 4000 rpm centrifugal force for 60,000 s: (a) US‐PB, (b) MF‐PB, (c) US‐DW, and (d) MF‐DW. DW, distilled water; MF, microfluidization; PB, phosphate buffer; US, ultrasonication.

Samples prepared in PB displayed a gradual increase in size for both techniques, from ≈140 nm initially to > 300 nm by 35 days, indicating Ostwald ripening and creaming over time. Ostwald ripening is the coarsening of larger droplets at the expense of smaller ones. This phenomenon suggests that oil molecules migrated from smaller droplets to larger ones through the surrounding continuous phase. Sedaghat Doost, Van Camp, et al. (2019) reported that nanoemulsions loaded with EO are particularly vulnerable to this phenomenon. This trend was less pronounced in samples prepared in DW, where the particle size initially increased and then decreased during storage, indicating oiling‐off. Visual inspection also confirmed this, showing coalescence and visible oiling‐off in both samples prepared in DW (Figure 1). Particle size distribution data (Figures 2 and 3) also showed a narrower distribution for the ultrasonication method, indicating a smaller PDI. PDI reflects the uniformity of particle size distribution. Values below 0.3 generally indicate a monodisperse system suitable for food and pharmaceutical applications (Danaei et al. 2018; Tamjidi et al. 2013). All formulations in this study demonstrated acceptable PDI values of 0.144 ± 0.006, 0.161 ± 0.004, 0.259 ± 0.090, and 0.225 ± 0.010 for US‐PB, MF‐PB, US‐DW, and MF‐DW, respectively. These results indicate that both ultrasonication and microfluidization effectively created uniform nanoemulsion, regardless of the dispersion medium. However, the opposite trend was seen in samples prepared in DW compared to PB. US‐DW exhibited the highest PDI, and US‐PB the lowest. PDI increased notably during storage of samples in DW. This finding aligned with that of Rajasekaran et al. (2024), who reported greater stability and a narrower particle size for the ultrasonicated shrimp oil nanoemulsion compared to the microfluidized nanoemulsion.

However, Jafari et al. (2007) reported that microfluidization resulted in a narrower distribution (lower span) compared to ultrasonication. In their study, ultrasonication was performed without cooling and on a large volume of 500 mL. In contrast, this study performed ultrasonication at low temperature for only a 30 mL sample. Therefore, optimization is necessary for each experiment.

Measuring the pH changes in all four samples showed a decrease from 7.0 ± 0.1 to 5.3 ± 0.1 in the samples prepared with DW, while the samples prepared with PB maintained a stable pH of 7.0 ± 0.1. This difference might have caused an interfacial layer composed of lecithin rearrangement, leading to coalescence and oiling‐off. Furthermore, pH can influence this process by potentially speeding up structural changes (Roy et al. 2016).

Similar observations have been reported in previous studies, in which emulsion stability strongly depended on lecithin ionization. Tabaniag et al. (2023) demonstrated that altering the system pH changes the ionization of soybean lecithin's amino and phosphate groups, thereby influencing droplet charge, zeta potential, and electrostatic repulsion at the oil–water interface. Likewise, Pichot et al. (2013) found that lowering the pH reduces the zeta potential of emulsions, leading to coalescence and phase separation. These findings corroborate our results, suggesting that pH‐induced charge neutralization and molecular rearrangement contributed to the observed instability in the DW‐prepared samples.

### 3.2 Zeta Potential

Zeta potential provides insights into the surface charge of nanoemulsions and their electrostatic stability. The stronger the magnitude of the surface charge, either positive or negative, the greater the electrostatic repulsion, and the lower the tendency for droplets to aggregate (Naeem et al. 2015; Tamjidi et al. 2013). The absolute value of zeta potential 30 ≤ ζ ≤ 60 mV is considered excellent stability of the system (Jazani et al. 2022).

**FIGURE 4:**
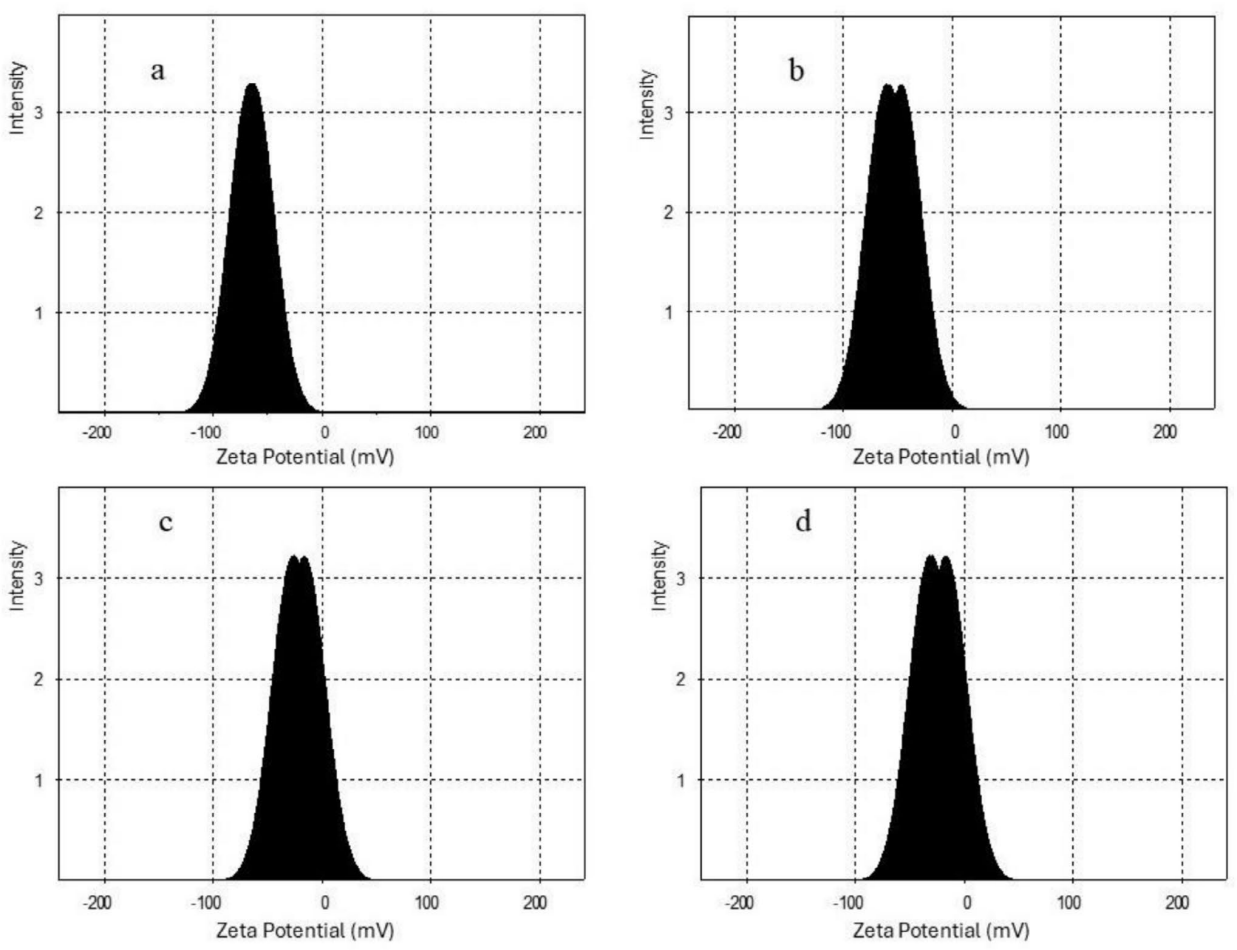
Zeta potential distribution of freshly prepared nanoemulsions (time zero): (a) US‐PB, (b) MF‐PB, (c) US‐DW, and (d) MF‐DW. DW, distilled water; MF, microfluidization; PB, phosphate buffer; US, ultrasonication.

In this study, nanoemulsions dispersed in PB exhibited high negative charges (Figure 4), with zeta potential of −56.89 ± 1.61 and −50.16 ± 5.34 mV for US‐PB and MF‐PB, respectively. These values indicate strong electrostatic repulsion and suggest that the system is electrostatically stable. These results are consistent with the report by Rajasekaran et al. (2024) on shrimp oil nanoemulsions, where the zeta potential of ultrasonicated nanoemulsions was higher than that of microfluidized ones.

In contrast, nanoemulsions in DW displayed moderately negative zeta potentials of −26.17 ± 7.90 and −22.93 ± 5.68 mV for MF‐DW and US‐DW, respectively. PB formulations are inherently more stable electrostatically and maintained at a stable pH, as discussed before. The reduced surface charge in samples prepared in DW led to unstable particle‐size evolution with altered growth and reduction, resulting in phase separation during storage.

### 3.3 Creaming Behavior and Physical Stability

The Lumisizer is a multi‐sample analytical centrifuge that enables accelerated stability testing of dispersions by simultaneously applying high centrifugal forces and measuring near‐infrared light transmission along the entire sample height. In this technique, changes in transmission over time are converted into space‐ and time‐resolved separation profiles, allowing quantitative evaluation of phenomena such as creaming, sedimentation, and clarification. By monitoring these profiles, key stability parameters, including creaming and instability index, can be extracted, providing a sensitive and rapid assessment of nanoemulsion physical stability under conditions that mimic long‐term storage.

**FIGURE 5:**
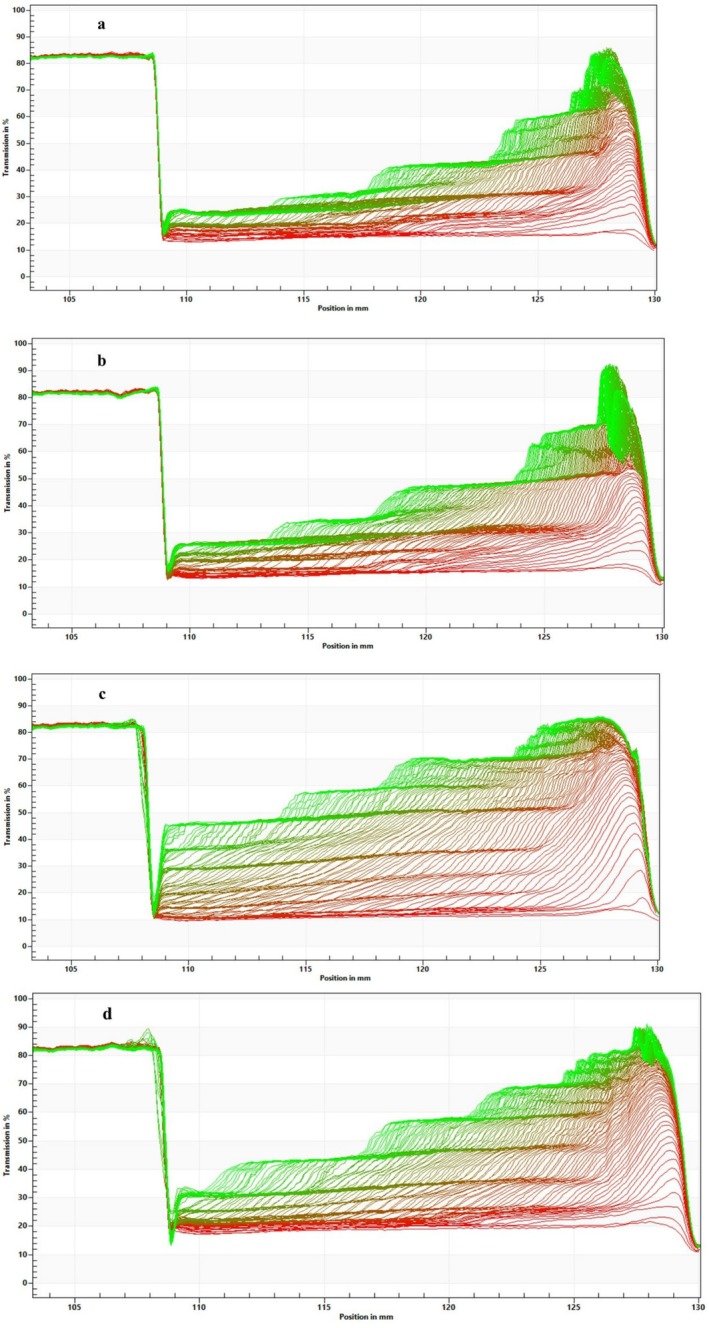
Creaming transmission profiles of samples under 4000 rpm centrifugal force during 120 min: (a) US‐PB, (b) MF‐PB, (c) US‐DW, and (d) MF‐DW. First profiles at the bottom; green lines: Last profiles in the top. DW, distilled water; MF, microfluidization; PB, phosphate buffer; US, ultrasonication.

Lower transmission (%) indicates greater creaming resistance. In this study, transmission profiles (Figure 5) reveal that DW samples exhibited greater shifts, indicating faster creaming and potential aggregation. In contrast, PB samples maintained more compact and overlapping profiles, confirming their resistance to phase separation. At 125 mm, the DW samples exhibited over 80% transmission, suggesting localized droplet depletion and creaming. In contrast, the US‐PB and MF‐PB samples showed 60% and 65% transmission, respectively. At a tube position of 115 mm, US‐PB showed the lowest transmission of 30%, indicating better stability of these samples compared to MF‐PB, which had a transmission of 35%, while US‐DW had 57%, and MF‐DW had 45%.

**FIGURE 6:**
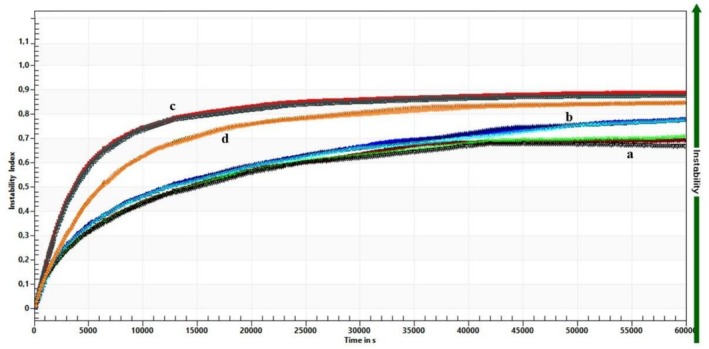
Instability index at centrifugal 4000 rpm during 60,000 s: (a) US‐PB, (b) MF‐PB, (c) US‐DW, and (d) MF‐DW. DW, distilled water; MF, microfluidization; PB, phosphate buffer; US, ultrasonication.

Stability was also quantified using the stability index, which ranges from 0 (high stability) to 1 (complete phase separation). The instability index (Figure 6) further confirmed these trends during 60,000 s under 4000 rpm: steeper slopes correspond to faster creaming and lower stability. The average of the final instability indices was 0.37, 0.40, 0.67, and 0.54 for US‐PB, MF‐PB, and MF‐DW, respectively.

Generally, samples prepared in PB, especially the ultrasonicated sample, demonstrated better long‐term physical stability. In contrast, samples prepared in DW, also notably ultrasonicated, showed less resistance to creaming. This could be caused by the lower zeta potential of US‐DW, leading to reduced electrical repulsion and creaming.

**FIGURE 7:**
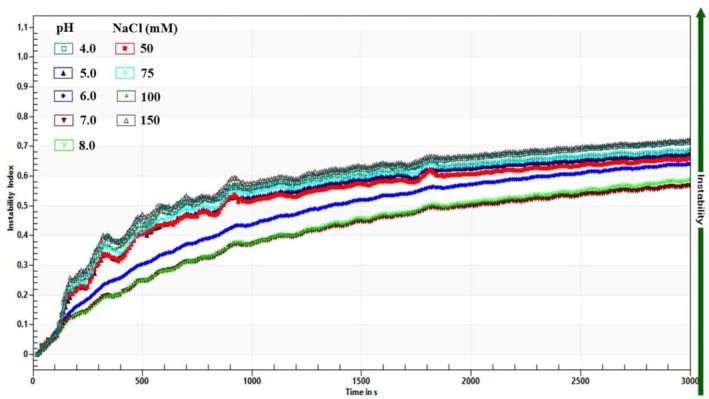
Instability index of US‐PB under different pH and ionic strength (4000 rpm during 3000 s).

US‐PB, known for its high stability, was tested under various pH and ionic strength conditions. According to colloidal stability theory, increasing NaCl concentration from 50 to 150 mM gradually reduced electrostatic repulsion between droplets, evidenced by the zeta potential becoming less negative (from −30.08 to −17.74 mV) and the instability index rising from 0.65 to 0.71 (Figure 7). This indicates that at high ionic strength, diminished electrostatic repulsion cannot counteract attractive van der Waals forces, leading to droplet aggregation, creaming, and phase separation, aligning with research on salt‐induced nanoemulsion destabilization (Barekat et al. 2024; Hassanzadeh et al. 2022). pH and ionic strength can significantly change the absolute value of zeta potential (Jazani et al. 2022).

US‐PB with initial zeta potential −56.89 mV, an instability index of 0.56 (under centrifugal force of 4000 rpm for 120 min), was adjusted across pH values from 4.0 to 8.0. At pH 4.0, the system was most unstable, with the zeta potential rising toward less negative values (around −22.88 mV) and the instability index increasing to about 0.71. At pH 8.0, the zeta potential remained strongly negative (−53.69 mV), and the instability index was low (0.58), indicating stability similar to the original sample at pH 7.0.

The destabilization at pH 4 is due to protonation, which reduces the surface charge density and the magnitude of the zeta potential. As the zeta potential becomes less negative, electrostatic repulsion weakens, allowing van der Waals forces to dominate, promoting flocculation and coalescence, and increasing the instability index. Similar pH‐dependent effects have been observed in food and bioactive nanoemulsions, where lowering the pH toward or below the pKa or isoelectric point reduces turbidity stability and accelerates phase separation (Hassanzadeh et al. 2022). At pH 8.0, deprotonation of interfacial groups keeps a large negative zeta potential and strong electrostatic repulsion, preventing droplet aggregation and maintaining low instability indices.

### 3.4 EE

EE is a key indicator of the ability of the nanoemulsion system to effectively entrap and retain EOs, which are inherently volatile and chemically unstable. In this study, all VEO nanoemulsions exhibited relatively high EE values, confirming the suitability of lecithin‐based nanoemulsions as delivery systems. In this study, nanoemulsions prepared in PB showed significantly higher EE compared to those prepared in DW. The highest EE was observed for US‐PB (88.14%), followed by MF‐PB (85.41%). In contrast, samples prepared in DW exhibited lower EE values of 69.95% and 69.39% for US‐DW and MF‐DW, respectively.

The superior EE observed in formulations prepared in PB can be attributed to the buffering capacity and ionic composition of PB, which resulted in higher negative zeta potential measured for those formulations, indicating stronger electrostatic repulsion and thereby enhancing interfacial stability (Jazani et al. 2022). In contrast, the lower EE values in formulations prepared in DW are likely related to reduced electrostatic stabilization and pH fluctuations during storage, which may promote phospholipid rearrangement and partial release of incorporated oil into the aqueous phase (Roy et al. 2016).

When comparing the production methods, ultrasonication resulted in slightly higher EE than microfluidization in both dispersion media. This trend may be associated with the smaller droplet size and narrower size distribution produced by ultrasonication, which enhances oil‐lipid interactions and retention within the nanoemulsion structure. Overall, these results demonstrate that while both emulsification techniques are effective, the dispersion medium plays a dominant role in determining EE, with formulations prepared in PB using US offering the most efficient entrapment of VEO.

## 4 Conclusion

This study showed that both ultrasonication and microfluidization are effective high‐energy techniques for creating VEO‐loaded nanoemulsions with nanoscale droplet sizes and acceptable uniformity. Ultrasonication consistently produced smaller droplets, and lightly narrower size distribution medium, under the conditions used, was the main factor influencing stability: nanoemulsions prepared in phosphate buffer had highly negative zeta potentials (≈−56 mV), higher EE (> 85%), and better resistance to creaming compared to systems made with distilled water, which showed moderate surface charge, lower EE (≈69%), and greater susceptibility to phase separation. Among all formulations, the US‐PB nanoemulsion had the highest EE (88.14%) and overall stability. It is acknowledged that increasing the number of passes during microfluidization could further reduce droplet size; however, this would require substantially longer processing times.

Accelerated stability testing and environmental stress evaluations confirmed that electrostatic interactions primarily determine nanoemulsion stability. Increasing ionic strength from 50 to 150 mM NaCl gradually reduced the surface charge and made the system more unstable, demonstrating salt‐caused screening of electrostatic repulsion. Similarly, acidic conditions (pH 4.0) led to significant destabilization due to protonation and decreased surface charge, while alkaline conditions (pH 8.0) maintained a strong zeta potential and low instability index. These findings emphasize that optimal nanoemulsion performance depends on controlling the production process, dispersion medium, EE, and environmental factors, with ultrasonicated PB‐formulation providing the most reliable approach for incorporating VEO under the conditions used in this research.

## Author Contributions

**Paul Van der Meeren:** conceptualization, writing – review and editing, supervision, methodology, validation. **Ali Sedaghat Doost:** conceptualization, methodology, validation. **Azizeh Rezaei:** investigation, validation, writing – original draft, writing – review and editing. **Zohreh Hamidi‐Esfahani:** conceptualization, writing – review and editing, supervision.

## Ethics Statement

The authors have nothing to report.

## Conflicts of Interest

The authors declare no conflicts of interest.

## Data Availability

The data that support the findings of this study are available on request from the corresponding author. The data are not publicly available due to privacy or ethical restrictions.
